# Oligonucleotide Therapeutics: From Discovery and Development to Patentability

**DOI:** 10.3390/pharmaceutics14020260

**Published:** 2022-01-22

**Authors:** Lara Moumné, Anne-Céline Marie, Nicolas Crouvezier

**Affiliations:** Inserm Transfert, Paris Biopark, 7 rue Watt, 75013 Paris, France; anne-celine.marie@inserm-transfert.fr (A.-C.M.); nicolas.crouvezier@inserm-transfert.fr (N.C.)

**Keywords:** oligonucleotides, antisense, small interfering RNA, exon skipping, small activating RNA, microRNA, nucleic acid targeting, patentability, protection, intellectual property

## Abstract

Following the first proof of concept of using small nucleic acids to modulate gene expression, a long period of maturation led, at the end of the last century, to the first marketing authorization of an oligonucleotide-based therapy. Since then, 12 more compounds have hit the market and many more are in late clinical development. Many companies were founded to exploit their therapeutic potential and Big Pharma was quickly convinced that oligonucleotides could represent credible alternatives to protein-targeting products. Many technologies have been developed to improve oligonucleotide pharmacokinetics and pharmacodynamics. Initially targeting rare diseases and niche markets, oligonucleotides are now able to benefit large patient populations. However, there is still room for oligonucleotide improvement and further breakthroughs are likely to emerge in the coming years. In this review we provide an overview of therapeutic oligonucleotides. We present in particular the different types of oligonucleotides and their modes of action, the tissues they target and the routes by which they are administered to patients, and the therapeutic areas in which they are used. In addition, we present the different ways of patenting oligonucleotides. We finally discuss future challenges and opportunities for this drug-discovery platform.

## 1. Introduction

The development of new drugs requires two major steps: the identification of a therapeutically relevant target and the development of a compound capable of modulating its function. Over the past century, drug development efforts were focused on targeting proteins with different types of compounds including small molecules and monoclonal antibodies. Many drugs have thus been developed for the treatment of a large spectrum of pathologies and, to date, protein targeting remains a privileged avenue in drug discovery. However, the development of a compound capable of inhibiting or activating the function of a protein requires the recognition of its complicated spatial conformation. Although some classes of proteins such as membrane receptors, enzymes, ion channels, or transport proteins can be therapeutically approached using conventional protein-targeting strategies, other targets like transcription factors, scaffold proteins, or structural proteins are much less druggable using traditional modalities [1].

An alternative to modulating the function of a protein is to modulate its expression level, and this can be achieved by acting on its mRNA (messenger ribonucleic acid). Oligonucleotides are a class of single- or double-stranded small synthetic nucleic acid polymers (≈20-mer) that can be used to modulate gene expression [2]. In this review, we focus on oligonucleotides designed to bind to RNA (ribonucleic acid) or DNA (deoxyribonucleic acid) by Watson–Crick base pairing. Oligonucleotides act on gene expression via various mechanisms. They can target pre-mRNA, mRNA, or non-coding RNA to induce degradation, modulate splicing events, or interfere with protein translation. Transcriptional activation can also be achieved using a specific class of oligonucleotide called small activating RNA (saRNA) through direct interaction with gene promoters [3]. Since they execute their function by complete Watson–Crick base pairing with DNA or RNA, oligonucleotides can in theory target any gene of interest since only the right nucleotide sequence along the targeted DNA or RNA needs to be selected. This considerably expands the number of proteins that can be targeted through the modulation of their mRNA expression. In addition, non-coding RNA, including microRNA (miRNA or miR) or long non-coding RNA (lncRNA), which are emerging as potential therapeutic targets, can also be modulated by oligonucleotides [4]. Furthermore, since the action of oligonucleotides requires high complementarity with the target sequence, oligonucleotides should, in principle, be much more specific than small molecule drugs.

The use of oligonucleotides as therapeutic agents was first proposed in the late 1970s [5], but delivery, stability, and specificity issues had to be solved to allow their medical use. Extensive research programs aimed at chemically optimizing oligonucleotides have been undertaken. Modifications of the phosphodiester bonds and of the sugar groups have been developed to improve oligonucleotide stability in plasma by increasing their resistance to nucleases and their affinity for serum proteins as well as their specificity for their target sequence [2]. Formulations and conjugations with specific chemical groups were developed to overcome delivery limitations and tissue specificity [2].

After having experienced ups and downs, the field of therapeutic oligonucleotides is now rapidly growing. Since the first approval of an oligonucleotide for medical use in 1998 [6], 12 other oligonucleotides have hit the market and a broad pipeline is currently in late clinical development. One of the marketed oligonucleotides, nusinersen/Spinraza [7,8], has been a commercial success, validating the commercial potential of oligonucleotide drugs. To date, 44 companies have molecules on the market or in late clinical development (past phase II). These include oligonucleotide-focused biotech companies like Ionis Pharmaceuticals and Alnylam Pharmaceuticals as well as Big Pharma companies like Johnson & Johnson, Roche, Novartis, and AstraZeneca. Paralleling the emergence of therapeutic oligonucleotides, there has been important activity relating to patent applications and patents in the field of oligonucleotides.

The aim of this review is to provide an overview of therapeutic oligonucleotides on the market or in advanced clinical development. We present, in particular, the different types of oligonucleotides and their modes of action, the tissues they target, and the routes by which they are administered to patients, as well as the therapeutic areas in which they are used. In addition, we present the different ways of patenting oligonucleotides and how two of the world’s leading patent offices assess inventions relating to oligonucleotides.

## 2. Oligonucleotides on the Market and in Clinical Development

We searched GlobalData, Clinicaltrials.gov (accessed on 20 November 2021), and PubMed for pertinent information on oligonucleotide drugs that target gene expression via direct interaction with RNA or DNA, with a focus on compounds that have hit the market or are in advanced stages of clinical development (phases II and III). We identified 93 compounds that meet these criteria. Among these oligonucleotides, 13 obtained approval between 1998 and 2021 and one is under regulatory review for approval (preregistration). One hundred and thirty phase II or phase III clinical trials involving 80 oligonucleotides are ongoing. These oligonucleotides are approved or tested in 102 different indications covering 14 therapeutic areas. They target 66 different genes.

### 2.1. Type of Olignucleotides and Mode of Action

#### 2.1.1. Antisense Oligonucleotides (ASOs)

**Figure 1:**
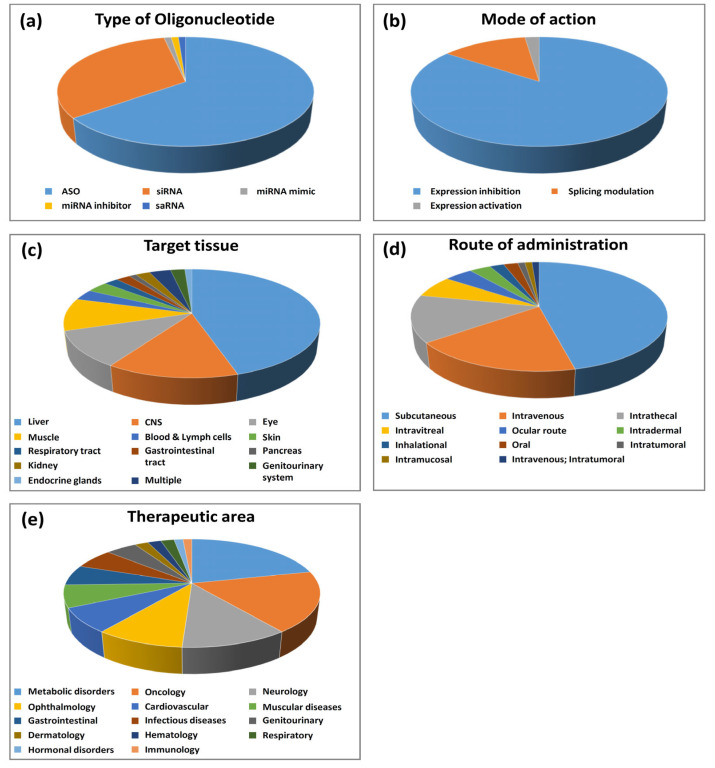
Statistical analysis of oligonucleotides on the market and in clinical development. (**a**) Type of oligonucleotide; (**b**) mode of action; (**c**) target tissue; (**d**) route of administration; (**e**) therapeutic area.

The most represented oligonucleotides in our analysis are antisense oligonucleotides (ASOs) (see Figure 1a). They account for about 65% of the total number and nine out of 13 oligonucleotides having obtained marketing authorization. ASOs are subdivided into two major groups according to their mode of action: gene-expression inhibitors (48 molecules) and splicing modulators (12 molecules). Expression inhibitors act via a mechanism involving RNAse H [9]. This enzyme binds to DNA/RNA duplexes to degrade the RNA strand. To act through this mechanism, expression inhibitors should have a DNA-like chemical structure. Some chemical modifications have been developed to improve the properties of these molecules without preventing their ability to induce RNAse H activity. The splicing modulators are designed to bind to the intron–exon junctions of pre-mRNAs where they induce steric hindrance to prevent splicing events [9]. Most splicing modulators have a DNA-like chemical structure, but chemical modifications were developed to prevent RNAse H recognition of the duplexes formed between the pre-mRNAs and the splicing inhibitors. Some splicing inhibitors have an RNA-like chemical structure. All the ASOs identified in our analysis are listed in Table 1.

- Gene expression-inhibiting ASOs

Among the gene-expression inhibitors, four have received marketing authorization, and 44 are currently in phase II or phase III clinical trials.

**Figure 2:**
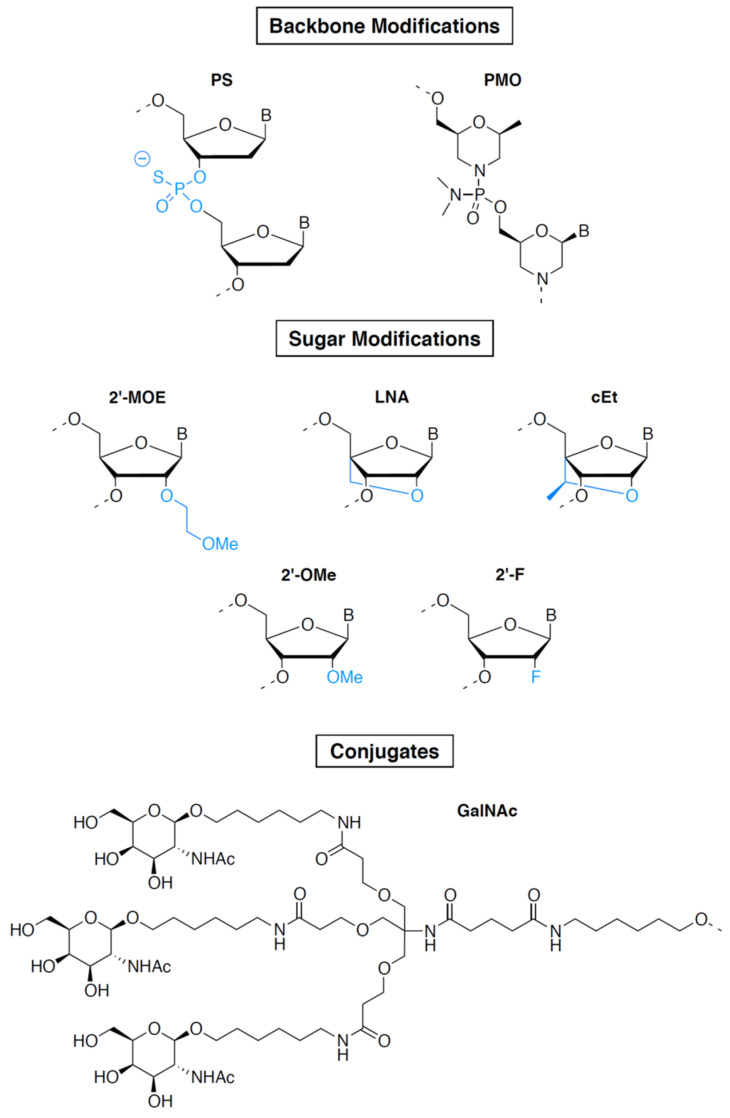
Common chemical modifications used in oligonucleotide drugs. Upper panel: modifications of the phosphate backbone. Middle panel: modifications of the 2′ position of the sugar. Lower panel: targeting ligand. Abbreviations: PS, phosphorothioate; PMO, phosphorodiamidate morpholino oligomer; 2′-MOE, 2′-O-methoxyethyl; LNA, locked nucleic acid; cEt, (S)-constrained ethyl nucleic acid; 2′-OMe, 2′-O-methyl; 2′-F, 2′-Fluoro; GalNAc, *N*-acetylgalactosamine.

The first ASO to be approved in 1998 was fomivirsen/Vitravene, an ASO drug targeting the cytomegalovirus (CMV) RNA sequence for the treatment of CMV retinitis in immunocompromised patients, including those with acquired immunodeficiency syndrome (AIDS) [6]. However, the drug was withdrawn because of the high medical need that existed at the time the drug was discovered and developed due to CMV arising in AIDS patients, which dramatically decreased a few years later due to human immunodeficiency virus (HIV) triple therapy. Initially discovered at the National Institutes of Health (NIH), it was licensed and developed by Isis Pharmaceuticals (now Ionis Pharmaceuticals), which subsequently licensed it to Novartis. Fomivirsen contains phosphorothioate (PS) bonds in which a non-bridging oxygen atom is replaced by sulfur in the phosphodiester bonds connecting the nucleosides (Figure 2) [10,11]. Most gene expression-inhibiting ASOs contain this first-generation ASO modification, which increases the stability of ASOs in plasma by preventing their degradation by nucleases and by increasing their binding to plasma proteins, including albumin.

Fifteen years after this first access to the market by an oligonucleotide, a second ASO, mipomersen/Kynamro, was approved in 2013 for the treatment of homozygous familial hypercholesterolemia [12]. Mipomersen is a second-generation ASO. It contains, in addition to PS linkages, 2′-O-methoxyethyl (2′-MOE)-modified riboses (see Figure 2) at both the 5′ and 3′ ends of the molecule (on five nucleotides at each end) and unmodified deoxyribonucleotides in the middle part of the molecule (gap region of 10 nucleotides) [11]. 2′-MOE makes the drug more resistant to degradation by nucleases, allowing weekly administration. In addition, 2′-MOE improves binding affinity to the target RNA and reduces toxicity due to non-specific protein binding. When used in a fully modified ASO, 2′-MOE inhibits the activity of RNAse H. The “gapmer” pattern combining unmodified nucleotides and 2′-MOE takes advantage of the intrinsic properties of 2′-MOE without affecting the capacity of the ASO to induce degradation of its target RNA, making this chemical design perfectly suited for gene-expression inhibition [11]. It was extensively used thereafter and is found in two additional marketed ASOs: inotersen/Tegsedi [13] and volanesorsen/Waylivra [14], which respectively target TTR and APOC3 genes and have been approved for the treatment of familial amyloid neuropathies and familial chylomicronemia, respectively. These two molecules were developed by AKCEA Therapeutics, an affiliate of Ionis Pharmaceuticals. Our analysis shows that at least 26 ASOs in phase II or phase III clinical trials also contain PS/2′-MOE gapmers. Interestingly, at least eight of these molecules have their targets in the central nervous system (CNS). Targeting the CNS with 2′-MOE-containing ASOs is facilitated by their high stability in the cerebrospinal fluid (CSF) after intrathecal injection, which makes them particularly suitable for CNS targeting [15].

Two other modifications used in gapmer ASOs are currently in phase II clinical trials: (S)-constrained ethyl (cET) and locked nucleic acid (LNA) (Figure 2) [11]. AZD-8701, danvatirsen, and IONISAR-2.5Rx are the most advanced cET-modified ASOs and are currently being evaluated in phase II clinical trials. AZD-8701 and danvatirsen are PS/cET gapmer ASOs developed by AstraZeneca. They target the FOXP3 and STAT3 genes, respectively, and are being evaluated for treatment of different types of cancer [16,17]. IONISAR-2.5Rx is a PS/cET gapmer ASO developed by Ionis Pharmaceuticals and designed to inhibit the expression of the androgen receptor (AR gene) for the treatment of castration-resistant prostate cancer [18]. Two LNA gapmers are currently in phase II clinical trials: cepadacursen, an ASO targeting PCSK9 expression for the treatment of hypercholesterolemia [19], and ISTH-0036, an ASO targeting TGFB2 expression for the treatment of diabetic macular edema and wet macular degeneration [20]. LNA and cET have a methylene bridge connection between 2′-oxygen and the 4′-carbon of the ribose, constraining the base into a conformation predominantly characterizing the RNA ribose sugar and preventing the conformation characteristic of the DNA ribose sugar [21]. This results in an increase in specificity and affinity for the target and a reduction in recognition by nucleases. Like 2′-MOE, LNA and cET do not hamper the activity of RNAse H only when used as gapmers. Despite their chemical properties, which make them powerful tools for gene-expression inhibition, clinical development of cET and LNA gapmers has been hampered by the risk of hepatotoxicity, and to date, 2′-MOE gapmers are much more widely used.

One of the main limitations of using ASOs as drugs is their poor cell penetration and lack of tissue specificity. Several technologies overcoming these limitations are under development. In particular, Ionis Pharmaceuticals embarked on a development program aimed at generating ligand-conjugated ASO (LICA) in order to direct a larger fraction of a dose to desired tissues and improve both cellular uptake and specific tissue targeting. Ionis Pharmaceuticals first used a technology developed by Alnylam consisting of conjugating *N*-acetylgalactosamine (GalNAc) to an oligonucleotide (see Figure 2 and the siRNA section) for liver-specific targeting [22,23]. To date, at least 12 GalNAc-conjugated ASOs are in phase II or phase III clinical trials. These include eplontersen [24] and olezarsen [25], which are GalNAc-conjugated versions of two marketed drugs mentioned above, Inotersen/Tegsedi and Volanesorsen/Waylivra, respectively.

- Splicing–modifying ASOs

The second major group of ASOs consists of splicing modulators. To date, five splicing–modulating ASOs have received marketing authorization, and seven are in phase II or phase III clinical trials. All these compounds target rare genetic diseases.

The best-known ASO in this group is nusinersen/Spinraza, which in December 2016 became the first approved drug used in the treatment of spinal muscular atrophy (SMA) [8]. This rare genetic neuromuscular disease, characterized by loss of motor neurons and progressive muscle decline, is caused by the mutations of the survival of motor neuron 1 (SMN1) gene, which results in the loss of survival of motor neuron (SMN) protein function [26]. SMN1 has a paralog gene, survival of motor neuron 2 (SMN2), that is nearly identical but undergoes an alternative splicing, resulting in exon7 skipping due to a variation in a single nucleotide (840C→T). This alternative splicing results in only 10% to 20% of SMN2 transcripts coding a fully functional SMN and 80% to 90% of transcripts resulting in a truncated protein (SMNΔ7) that is rapidly degraded in the cell [27]. Nusinersen/Spinraza was designed to target a specific sequence in the intron downstream of SMN2 exon7 in order to modify the alternative splicing and to induce a retention of exon7 in the transcripts. This leads to the production of a higher percentage of full-length SMN protein. nusinersen/Spinraza is a fully modified PS, 2′-MOE ASO administered intrathecally once every two weeks to children with SMA, leading to improved motor functions and prolonged survival [7]. Having initially developed nusinersen/Spinraza in collaboration with Cold Spring Harbor Laboratory, Ionis Pharmaceuticals partnered in development with Biogen, who acquired an exclusive license for the drug. After its marketing authorization in 2016, nusinersen/Spinraza quickly became a blockbuster and makes about 2 billion dollars in sales per year. However, this success is likely to be mitigated by the market access of two competing drugs: onasemnogene abeparvovec/Zolgensma [28] and risdiplam/Evrysdi [29] approved in May 2019 and August 2020, respectively. Zolgensma is a gene therapy based on the overexpression of SMN1 developed by AveXis, a US biotechnology startup acquired by Novartis in 2018. Risdiplam/Evrysdi is a small molecule whose mechanism of action is similar to that of Spinraza since it acts as a splicing modifier of SMN2 gene. Evrysdi was developed by PTC Therapeutics in association with the Spinal Muscular Atrophy (SMA) Foundation and is marketed by Roche/Genentech. It is the first oral medication approved to treat SMA.

Another CNS disease-targeting splicing modulator, STK-001, is currently being developed by Stoke Therapeutics for the treatment of Dravet syndrome, an autosomal dominant genetic disorder that causes a very severe form of epilepsy [30]. STK-001 is designed to upregulate the expression of the Nav1.1 protein that is encoded by the sodium channel, voltage-gated, type I, alpha subunit (SCN1A) gene, the mutations of which are responsible for a reduction in the expression of Dravet syndrome.

Splicing modulation-based approaches have been the most widely explored for Duchenne muscular dystrophy (DMD), the most common form of inherited myopathies [31]. To date, four ASOs targeting splicing events in the DMD gene have received marketing authorization and four are in phase II clinical trials. DMD is caused by mutations in the DMD gene, the longest gene in the human genome, which contains 79 exons and encodes the dystrophin protein. A wide spectrum of mutations, including deletions, duplications, insertions, and point mutations, have the potential to disrupt the reading frame and produce premature termination of translation, leading to complete loss of the dystrophin protein. The majority of these mutations are located at a major mutational hotspot encompassing exons 43–55 [32]. Exon skipping has emerged as a potential therapy for DMD patients, the objective being to eliminate a mutation-containing exon from the mature mRNA to reframe the dystrophin transcript giving rise to a partially deleted yet functional dystrophin protein [31]. There are several research and development programs currently aimed at targeting exons of the so-called major mutational hotspot. With the aim of developing a personalized medicine approach, Sarepta Therapeutics currently has in its pipeline eight ASOs designated to induce the skipping of specific exons from this region at development stages ranging from discovery to commercialization, among which are three FDA-approved drugs: eteplirsen/Exondys 51 (exon51), golodirsen/Vyondys 53 (exon53) and casimersen/Amondys 45 (exon45) [33,34,35]. Recently, another ASO targeting exon53 received marketing authorization: viltolarsen/Viltepso developed by NS Pharma, a subsidiary of Nippon Shinyaku [36]. The target region of viltolarsen is the same as that of golodirsen, but the sequence is four nucleotides shorter. Eteplirsen, golodirsen, casimersen, and viltolarsen have been approved on the basis of their ability to increase the production of dystrophin. However, a substantial and clear clinical benefit remains to be demonstrated. These ASOs are based on phosphorodiamidate morpholino oligomer (PMO) chemistry and consist of DNA bases attached to a backbone of methylene–morpholine rings linked through phosphorodiamidate groups (Figure 2) [11]. PMO ASOs hybridize to their target RNA and block access to other molecules (steric hindrance) without inducing RNA degradation. They are particularly suitable for mechanisms of action that do not involve RNAse H, such as splicing modulation. PMOs display a high stability and excellent safety but their low protein binding results in poor pharmacokinetics and limited potency. To overcome these limitations, efforts have been made to increase cellular uptake of PMOs through the development of cell-penetrating peptide-conjugated PMO oligomers (PPMOs) [37]. PPMOs are currently developed by Sarepta Therapeutics as next-generation ASO-based therapies. Nonclinical studies have demonstrated enhanced and targeted delivery to skeletal, cardiac, and smooth muscle cells, as well as subsequent increased mRNA modification and dystrophin production. Six PPMOs are currently being developed by Sarepta, among which the most advanced is SRP-5051, the PPMO derivative of eteplirsen, which is currently being evaluated in a phase II clinical trial.

Two exon-skipping ASOs developed by ProQR are being evaluated in clinical trials in genetic ophthalmologic diseases. Sepofarsen, an RNA antisense oligonucleotide that aims at restoring vision in Leber congenital amaurosis patients with the most common p.Cys998X mutation in the CEP290 gene, is currently in phase III [38]. QRX-421a aims at preventing vision loss or restoring vision in retinitis pigmentosa (RP) and Usher syndrome type 2 patients carrying mutations in USH2A gene exon 13 [39]. It is currently being evaluated in a phase II clinical trial.

#### 2.1.2. Small Interfering RNA (siRNA)

The second type of oligonucleotides most represented in our analysis are siRNAs. They account for about 32% of the total number (see Figure 1a) and four out of 13 oligonucleotides having obtained marketing authorization. In addition, one siRNA is currently in pre-registration and 25 are in phase II or phase III clinical trials. All the siRNAs identified in our analysis are listed in Table 2.

SiRNAs are double-stranded RNA molecules capable of hybridizing specifically to their target RNA via Watson–Crick base pairings [40]. Due to their mechanism of action utilizing the microRNA (miRNA) machinery to induce RNA degradation or translation inhibition, they all act as inhibitors of gene expression. In 2018, patisiran/Onpattro became the first marketed siRNA drug [41], 20 years after the discovery of RNA interference in 1998 [42]. In 2001, Elbashir et al. showed that chemically synthesized duplexes of 21-base-pair RNA were able to silence the expression of a specific gene in mammalian cells, establishing the first proof of concept of the use of siRNA as gene-expression inhibitors [43]. Together with ASOs, siRNAs then became promising tools for targeting the expression of proteins involved in pathogenic mechanisms, paving the way for the development of a new class of drugs. However, the development of siRNA as drugs was slowed down by three major obstacles: delivery, stability, and specificity [40]. The delivery limitation is due to siRNA’s high molecular weight (≈13 kilodaltons for a 20-base-pair siRNA, twice as much as ASOs) and their high negative charge, which prevents them from crossing cell membranes. Their lack of stability is due to both the phosphodiester bonds and the 2′-OH nucleophilic group on the ribose responsible for the hydrolysis of RNA. Although in theory siRNAs only degrade their target RNA when completely base paired, some mismatches are actually tolerated by the miRNA machinery, which can lead to off-target effects, causing specificity issues. The development of chemical modifications capable of removing these brakes has allowed the development of drugs based on siRNA. In particular, the use of phosphorothioate and modifications of the ribose 2′ position, like 2′-O-methyl (2′-OMe) or 2′-Fluoro (2′-F) (see Figure 2), were used to improve the stability and specificity, whereas formulations and conjugations have been used to overcome delivery limitations [11]. The most significant breakthrough was the use of GalNAc conjugates to improve siRNA cell penetration and specific targeting of the liver [22,23].

The aforementioned siRNA drug patisiran, which was designed to inhibit the expression of the TTR gene, gained marketing approval for the treatment of familial amyloid neuropathies in 2018 [41]. This siRNA, developed by Alnylam, contains a mixture of unmodified and 2′-O-methylated (2′-O-Me) ribonucleotides. This modification enhances siRNA stability by replacing the labile OH group on the ribose 2′ position with a stable OCH_3_ group [11]. This modification has been extensively used alone or in combination with other modifications to increase siRNA stability, specificity, and binding affinity. It is used in at least 14 out of 30 siRNAs either on the market, in pre-registration, or in phase II or phase III clinical trials. In addition to nucleotide modifications, patisiran was formulated in a lipid nanoparticle (LNP) to enhance its cellular uptake and delivery in the liver where its target gene (i.e., transthyretin (TTR)) is highly expressed. LNPs serve to mask the siRNA charges and facilitate both endocytosis and endosomal escape into the cytoplasm. In addition, LNPs enhance siRNA stability by protecting them from degradation by RNases. LNPs primarily accumulate in the liver due to their interaction with serum lipoproteins that interact with the low-density lipoprotein receptor (LDLR) on the surface of hepatocytes [44]. Patisiran is administered by intravenous infusion once every three weeks.

In 2019 a second siRNA developed by Alnylam, givosiran/Givlaari, was approved for the treatment of acute hepatic porphyria, a family of rare genetic diseases associated with hypomorphic mutations in genes encoding enzymes involved in heme synthesis [45]. These mutations lead to the accumulation of neurotoxic intermediate metabolites that cause neurovisceral attacks and chronic manifestations. This toxic metabolite accumulation can be prevented by the inhibition of aminolevulinic acid synthase, a hepatic enzyme encoded by the ALAS1 gene, the target of givosiran. Importantly, givosiran was the first marketed GalNAc conjugate. It is administered via subcutaneous injections once a month. GalNAc conjugates binds to the asialoglycoprotein receptor (ASGPR) that is highly expressed on the hepatocyte surface, resulting in rapid endocytosis of siRNA. Although the exact mechanism of escape across the endosomal membrane remains unknown, substantial amounts of siRNAs enter the cytoplasm to reach their target RNA and induce robust RNAi responses in vivo. Initially developed by Alnylam Pharmaceuticals, GalNAc conjugates are now developed by other siRNA biotech companies (like Dicerna Pharmaceuticals and Arrowhead Pharmaceuticals) and Big Pharma (like Sanofi and Novartis). Three out of four siRNAs on the market are GalNAc conjugates, including lumasiran/Oxlumo and inclisiran/Leqvio, which received approval for medical use in 2020 [46,47].

Lumasiran was developed by Alnylam for the treatment of primary hyperoxaluria type 1 (PH1), a rare genetic disorder caused by mutations of the AGXT gene. The loss of function of the AGXT-encoded liver enzyme alanine:glyoxylate aminotransferase causes the liver to produce an excessive amount of oxalate, which accumulates in the kidney and induces kidney stones and kidney failure, leading ultimately to multi-organ damage. Lumasiran was designed to reduce hepatic levels of hydroxy acid oxidase 1 (HAO1), depleting glyoxylate, the substrate necessary for oxalate production, and thus preventing its pathogenic accumulation in PH1 patients.

Inclisiran/Leqvio is a medication for the treatment of people with atherosclerotic cardiovascular disease (ASCVD) and hypercholesterolemia that was developed by The Medicines Company, which was acquired by Novartis in 2019. Inclisiran is a GalNAc conjugate that reduces the expression of liver proprotein convertase subtilisin/kexin type 9 (PCSK9). This protein plays a major regulatory role in cholesterol homeostasis, mainly by reducing LDLR levels on the plasma membrane, resulting in decreased metabolism of LDL particles, which can lead to hypercholesterolemia [48]. PSCK9 has been widely studied as a therapeutic target and various inhibitors have been developed, including monoclonal antibodies. However, the low frequency of administration of inclisiran (once every six months) [49] is an advantage over monoclonal antibodies, which are injected more frequently. Other companies are developing PCSK9-targeting oligonucleotides (ASOs), including AZD-8233 (AstraZeneca) and Cepadacursen (Civi Biopharma), which are currently in phase II clinical trials [19,50]. Interestingly, although all other marketed oligonucleotides (ASOs and siRNAs) target rare diseases, inclisiran is the first oligonucleotide drug to benefit a large patient population.

Among the 30 siRNAs either on the market, in pre-registration, or in phase II or phase III clinical trials, 19 are GalNAc conjugates. Together with the 12 GalNAc-conjugated ASOs mentioned above, GalNAc conjugates represent a third of the total oligonucleotides identified in our analysis (31 out of 93) and almost 75% of the oligonucleotides with their target in the liver (31 out of 42). Any gene with pathogenic dysregulated expression in the liver can therefore be considered a potential target for oligonucleotide-based therapy. There are only two siRNAs unconjugated to GalNAc and targeting the liver: patisiran (previously mentioned) and BMS-986263, developed by Bristol-Myers Squibb and currently being evaluated in a phase II clinical trial for liver fibrosis [51]. These two molecules are coupled to lipid nanoparticles, which are also good tools to achieve delivery in the liver. BMS-986263 is also being developed for idiopathic pulmonary fibrosis (phase II).

#### 2.1.3. Oligonucleotides Other Than ASOs and siRNAs

Among the 93 oligonucleotides in our analysis, only three are not ASOs or siRNAs: remlarsen and lademirsen, which act through mechanisms involving miRNAs and MTL-CEBPA, a small activating RNA (saRNA) (Figure 1a and Table 3).

MicroRNAs are small noncoding RNAs that function as important posttranscriptional regulators of gene expression [52]. They can either induce mRNA degradation or translation inhibition, thus downregulating their target genes. A single miRNA can silence the expression of a number of functionally related genes. Up- and downregulation of miRNAs has been associated with pathogenic pathways in several human diseases and therapeutic tools are being developed to mimic the activity or, on the contrary, decrease the expression of specific miRNAs. Remlarsen is an oligonucleotide developed by Miragen Therapeutics and designed to mimic the activity of miR-29, a miRNA that acts as a negative regulator of a wide variety of genes important in extracellular matrix deposition. MiR-29 expression is reduced in pathological fibrotic conditions. By mimicking the activity of miR-29, remlarsen decreases collagen expression, thereby ameliorating the disease condition [53]. Remlarsen is being developed for a wide range of pathogenic conditions involving fibrotic processes in several therapeutic areas, including cardiovascular, ophthalmology, respiratory, and dermatology, and is being evaluated in a phase II clinical trial for the treatment of keloids. It has a DNA chemical structure with LNA sugar modifications. Lademirsen is a single-stranded RNA sugar-modified oligonucleotide that inhibits miR-21, a microRNA widely expressed in multiple cell types in the kidney [54]. Upregulation of miR-21 contributes to the pathogenesis of multiple acute and chronic kidney diseases. By inhibiting miR-21, lademirsen downregulates scarring and kidney damage. It is currently being evaluated in a phase II clinical trial for Alport syndrome, a genetic disorder characterized by chronic kidney disease known as glomerulonephritis, which inexorably progresses to end-stage kidney disease in young adults. Remlarsen was first developed by Regulus Therapeutics, who licensed the worldwide rights to the drug to Sanofi.

All the oligonucleotides mentioned above target transcripts (pre-mRNA, mRNA, or non-coding RNA) to regulate gene expression by acting post-transcriptionally on RNA degradation, splicing, or translation regulation. Small activating RNA (saRNA) is a distinct class of non-coding RNAs that act in a completely different way since they target selected sequences in gene promoters to induce gene activation at the transcriptional/epigenetic level by a process known as RNA activation (RNAa) [3]. SaRNAs are 21-nucleotide double-stranded RNAs with 2-nucleotide overhangs at both ends, a structure identical to siRNAs despite their opposite biological functions. Whereas siRNAs induce mRNA degradation via the RISC complex within the cytoplasm and a subsequent reduction in gene expression, saRNA action takes place within the nucleus and involves the recruitment of the transcriptional machinery on targeted gene promoters. This results in an induction of the transcription and an increase in gene expression. Since the discovery of RNAa in 2006, improvements in saRNA design, chemistry, and understanding of the biology have matured the way to apply RNAa to cure human diseases. MTL-CEBPA was the first saRNA to reach clinics [55]. Developed by Mina Therapeutics, MTL-CEBPA is designed to specifically upregulate endogenous CCAAT/enhancer-binding protein alpha (CEBPA), a leucine zipper protein that acts as a master regulator of liver homeostasis and multiple oncogenic processes, including cell cycle control, proliferation, and angiogenesis. CEBPA also regulates the characteristics of myeloid cells, influencing the functions of immune cells in blood and tumor microenvironments. After showing increased expression of CEBPA mRNA in white blood cells after administration in patients, MTL-CEBPA is about to enter in a phase II clinical trial for hepatocellular carcinoma caused by a hepatitis B and/or C infection. MTL-CEBPA is a SMARTICLES liposomal nanoparticle encapsulating a 21-mer duplex 2′ O-Me saRNA.

In the next sections we provide information on oligonucleotide target genes, target tissues, the routes by which they are administered to patients, and the therapeutic areas in which they are used.

### 2.2. Oligonucleotide Target Genes

Although membrane receptors and enzymes are classically the targets of small molecules, and extracellular proteins or membrane receptors can be modulated with monoclonal antibodies, certain types of proteins such as structural proteins or transcription factors are generally hardly “druggable” [1]. Since they act at the DNA or RNA level, oligonucleotides can alter gene expression regardless of the function of the proteins encoded by their target genes, thus providing new opportunities to develop drugs against therapeutic targets of interest. Thus, among the targets of oligonucleotides, there are genes encoding proteins with a wide variety of functions, including classical drug targets (i.e., receptors and enzymes), as well as more challenging targets like structural proteins (e.g., dystrophin) and transcription factors (e.g., Forkhead box P3). In terms of mode of action, among the 93 oligonucleotides of our analysis, expression inhibitors (ASOs or siRNAs) represent 85% of the molecules (79 out of 93) and expression activators (splicing modulating ASOs, mir mimics, or saRNAs) represent 15% (see Figure 1b).

### 2.3. Target Tissues and Routes of Administration

We analyzed the oligonucleotides according to the tissues they target and their route of administration. Data are presented in Figure 1c,d. Interestingly, oligonucleotides can target all organs in the human body, but some tissues, mainly due to biological barriers, require specific routes of administration to be efficiently targeted. Therefore, although the majority of oligonucleotides are administered systemically (two thirds), mainly by a subcutaneous or intravenous route, a significant proportion of oligonucleotides are injected topically (one third). Unsurprisingly, the liver is the most represented tissue and is targeted by 46% of oligonucleotides. As mentioned before, targeting the liver is greatly facilitated by the development of chemical modifications and technologies such as GalNAcs and lipid nanoparticles. The vast majority of oligonucleotides targeting the liver, including all GalNAc conjugates, are injected subcutaneously. In addition to GalNAcs that allow specific liver targeting, subcutaneously injected oligonucleotides contain recurrent patterns of chemical modifications conferring high stability in the plasma after injection and a prolonged effect of the drug. For example, the vast majority of ASOs injected subcutaneously (20 out of 23) are PS/2′-MOE gapmers. Similarly, most siRNAs injected subcutaneously (17 out of 19) contain PS, as well as modifications of ribose 2′ position, like 2′-OMe or 2′F or a mixture of both. LNP-formulated oligonucleotides are injected intravenously. Muscle-targeted oligonucleotides, which represent about 10% of all oligonucleotides, are mostly injected intravenously, as they all target genetic diseases (mainly DMD) in which all muscles in the body need to be targeted. Common modification patterns used for intravenously injected oligonucleotides are PMOs or PS/cET gapmers for ASOs and 2′-OMe for siRNAs. To date, very few oligonucleotides are administered orally. In our study, only two compounds were reported to be administered orally in clinical trials for gastrointestinal diseases (alicaforsen and ION-253). Since these drugs target the gastrointestinal tract (GI), tablet formulations are used to topically deliver the products to sites of disease, with minimal systemic absorption. For example, a specific tablet was designed to release alicaforsen at the terminal ileum for delivery to the colon for the treatment of ulcerative colitis (see https://www.atlantichc.com/research/alicaforsen/, accessed on 5 January 2022).

The CNS is one of the most represented tissues in our analysis and is targeted by 14% of oligonucleotides. Due to the blood–brain barrier (BBB), the CNS cannot be reached after systemic administration such as subcutaneous or intravenous injections. Therefore, oligonucleotides targeting the CNS are all injected intrathecally into the CSF via lumbar puncture. The vast majority of CNS-targeting oligonucleotides (10 out of 14) contain PS and 2′-MOE modifications and exhibit unusual pharmacokinetic and pharmacodynamic properties, including complex active absorption mechanisms, low systemic exposure, extremely long half-lives, and accumulation and gradual release from subcellular deposits [15]. These properties greatly facilitated their clinical development, as illustrated by the market access of nusinersen/Spinraza, the first oligonucleotide approved for the treatment of a CNS disease. However, given the invasive nature of intrathecal administration, oligonucleotides are developed mainly for very severe neurological conditions with significant unmet medical needs and for which very few other therapeutic options exist. Further work on oligonucleotide uptake and development of formulations for delivery across the BBB are required for further optimization of the oligonucleotide drug development process for brain applications [15].

Like the CNS, the eye was one of the most represented organs in our analysis since it is targeted by 11% of oligonucleotides. Due to the blood–ocular barrier (BOB) that prevents drugs from traveling between the local blood vessels and most parts of the eye itself, eye targeting requires topical administration. This is a real advantage since the oligonucleotides administered in the eye do not pass into the bloodstream and only exert their effect locally, which reduces the side effects. Different routes of administration are used depending on the tissue to be reached: The ocular route via eye drop is mainly used to target the cornea, whereas targeting the retina requires more invasive intravitreal injections.

Other types of topical administrations can be used: Intradermal injections are used for skin targeting, whereas oligonucleotides can be inhaled for respiratory tract targeting.

### 2.4. Therapeutic Areas and Indications

Given the wide variety of tissues that can be targeted, oligonucleotide-based therapies can potentially be applied to all therapeutic areas and for many indications. The 93 oligonucleotides identified in our analysis are approved or tested in 102 different indications covering 14 therapeutic areas. With the exception of fomivirsen/Vitravene, which treated CMV retinitis, the first oligonucleotides were developed to treat genetic diseases. Of the 13 oligonucleotides that hit the market, 11 target rare genetic diseases. In many cases, the target gene of the oligonucleotide corresponds to the gene mutated in the disease. In 2020, inclisiran was the very first oligonucleotide drug benefiting a large population of patients with non-genetic disorders to be approved for medical use in metabolic and cardiovascular disorders. An increasing number of oligonucleotides is being developed for non-genetic diseases. To date, of the 102 indications found in our analysis, almost 75% are non-genetic diseases.

The distribution of the oligonucleotides according to the therapeutic areas is presented in Figure 1e. Consistent with the large number of hepatic targets, metabolic diseases are the most frequently targeted by oligonucleotides (21% in total and half of approved oligonucleotides). Indications in other therapeutic areas involving hepatic targets, like cardiovascular, gastrointestinal, and genitourinary (like lumasiran, which targets hepatic HAO1 to treat primary hyperoxaluria type I), were also frequently found in our analysis.

The second most represented therapeutic area is oncology (18%). Given that oncology is by far the therapeutic area most targeted by compounds developed by the pharmaceutical industry, the proportion of oligonucleotides in oncology remains relatively low. Only one phase III and eight phase II compounds are currently being evaluated in clinical trials and none has yet reached the market. In this therapeutic area, other modalities such as small molecules and monoclonal antibodies are still largely privileged.

Consistent with the pharmacokinetic and pharmacodynamic properties of oligonucleotides in the CSF described in the previous section, neurology is one of the main therapeutic areas for oligonucleotide therapeutics (11% in total and one approved oligonucleotide).

Thanks to the specific targeting of the eye due to the modes of administration, ophthalmology is also a therapeutic area of choice for oligonucleotides (9%). Although no oligonucleotide has yet received marketing authorization in this therapeutic area, five phase III clinical trials involving oligonucleotides are ongoing in ophthalmology [56].

Muscular disorders are targeted by 7% of total oligonucleotides and four out of 13 approved oligonucleotides. This is due to the very active research on Duchenne muscular dystrophy and in particular on the development of exon-skipping strategies to restore dystrophin function. Among the nine oligonucleotides developed for muscular diseases, only DYN-101, developed by Dynacure, targets another muscular pathology. DYN101 is designed to reduce the expression of dynamin 2 protein in several pathologies and is currently being evaluated in a phase II clinical trial for the treatment of X-linked myotubular myopathy [57].

Infectious diseases are also targeted by therapeutic oligonucleotides, in particular in the field of virology. As mentioned earlier, the first oligonucleotide to enter the market targeted CMV viral sequences for the treatment of CMV retinitis in immunocompromised patients [6]. Several oligonucleotides, including ASOs and siRNAs, target sequences of the hepatitis B virus (HBV) for the treatment of viral hepatitis [58,59,60]. These oligonucleotides target the liver and are mostly GalNAc conjugates. Interestingly, targeting viral sequences or cellular events related to viral infection by oligonucleotides can represent a therapeutic avenue for the development of new drugs to fight against viral infections, including the current COVID-19 pandemic. Several strategies can be developed: targeting either the virus itself by reducing the expression of its spike protein, or directly targeting its RNA genome with an ASO or siRNA. Alternatively, gene-silencing approaches can be used to reduce the inflammatory effects in the lungs and other organs that lead to mortality in severe cases of COVID-19. Several strategies are ongoing and the most advanced is donidalorsen. This ASO is designed to reduce the synthesis of prekallikrein (PKK), a precursor of the enzyme kallikrein, which subsequently inhibits bradykinin signaling and then inflammation. It is currently being evaluated in a phase II clinical trial for the management of respiratory complications in patients with severe forms of COVID-19. Donidalorsen is also undergoing a phase II clinical study in patients with hereditary angioedema (HAE) [2].

Other therapeutic areas, including dermatology, hematology, respiratory, endocrinology, and immunology, are also targeted by oligonucleotide therapeutics.

## 3. Patentability of Oligonucleotides

As discussed, great development of oligonucleotide therapeutics has been in progress for several decades by many actors and, to date, 44 companies have molecules on the market or in advanced clinical development (see Table 1, Table 2 and Table 3). The two market leaders are Ionis Pharmaceuticals for ASOs and Alnylam Pharmaceuticals for siRNAs. With its subsidiary AKCEA and its many partners (including Biogen, Roche, Novartis, Johnson & Johnson, and AstraZeneca), Ionis Pharmaceuticals has brought five ASOs to the market and currently has 34 molecules in phase II or III clinical development. Ionis Pharmaceuticals is involved in the development of more than 40% of the compounds described in our review. Pioneers in the development of GalNAc technology, Alnylam Pharmaceuticals, together with its partners, has four siRNAs on the market and seven others in phase II or III clinical development, most of them being GalNAc conjugates. Other companies, including Sarepta Therapeutics, which has three marketed ASOs and one in phase II, and Arrowhead Pharmaceuticals, which is currently evaluating five siRNAs in phase II clinical trials, are particularly active in the development of oligonucleotide therapeutics. This development is of course accompanied by significant activity by these companies in the field of intellectual property in an attempt to protect their innovations. In recent years, there has been an increase in the number of patent applications and patents related to oligonucleotides. For example, in the database of the World Intellectual Property Organization (WIPO: www.wipo.int, accessed on 20 November 2021), more than 89,000 references are identified with the code C12N 15/113, which corresponds to “Non-coding nucleic acids modulating gene expression, e.g., antisense oligonucleotides”.

The goal of a company trying to protect an innovation is to obtain the strongest and broadest scope of protection. However, depending on different aspects (e.g., eligibility, patentability, or the strategy of protection wishes) the drafting of the patent claims can differ, and the different patent offices may evaluate the invention differently. The purpose of this section is to provide an overview of the different types of protection that can be obtained and how the patent offices assess them.

### 3.1. Oligonucleotide Therapeutics: How to Patent Them?

Oligonucleotide therapeutics are generally used to (i) induce the degradation of an mRNA or non-coding RNA, (ii) inhibit the translation of an mRNA, (iii) induce splice switching, or (iv) activate the transcription of a gene (saRNA). For these purposes, inventors must design at least one oligonucleotide (i.e., a specific nucleic acid sequence) that will target a specific gene.

Thus, to obtain protection for a given oligonucleotide, the invention must be disclosed in a sufficiently clear and concise manner (=disclosure of the invention) and must also be new, involve an inventive step, and be capable of industrial application. An invention is new if it does not form part of the state of the art and involves an inventive step if, with regard to the state of the art, the solution given by the invention is not obvious to a person skilled in the art. If these two first criteria are analyzed in view of the state of art, the industrial application criterion is often easy to fulfill, because it is only necessary that the invention be made or used in any kind of industry, including agriculture.

Like many products, there are different strategies to protect oligonucleotides. For example, it is possible to protect the nucleic acid sequence of the oligonucleotide itself, a method of treatment based on the targeting of a gene or a region of an mRNA by an oligonucleotide or a new “format” (or scaffold) of an oligonucleotide. However, the patentability of this type of invention is judged differently according to the inventions and according to the patent offices.

The aim of the next part is to take stock of the practice in the field of oligonucleotide therapeutics and to explain the different strategies of protection and how the EP and US patent offices evaluate this kind of invention. The analysis of the novelty and inventive step criteria will not be discussed in detail here, but Table 4 gives some tips to help the future writer of a patent application apprehend them. Eligibility and disclosure of the invention (also known as sufficiency of description) to the EPO (European Patent Office) and USPTO (the United States Patent and Trademark Office) will be discussed below.

### 3.2. The Different Strategies of Oligonucleotide Therapeutics Protection

Oligonucleotides can be defined by a functional definition or by a structural definition. Indeed, when broad protection is requested and when the target of the oligonucleotide (the gene to be targeted) and its involvement in a pathology is new, a functional definition of the invention is possible. The writer will be able to define the oligonucleotide by its capacity to target the specific gene. Here is a theoretical example of a functional definition of a claim (e.g., see WO2006000057 [62] of Table 5 for a concrete example):

“An antisense oligonucleotide that reduces the expression and/or activity of [the target].”

This kind of patent application can also claim therapeutic methods using these oligonucleotides and the pharmaceutical composition containing them. Moreover, this kind of patent application generally claims specific oligonucleotides (specific nucleic acid sequences; see below) to protect the product as such and to provide fallback positions if the claim with the functional definition is not accepted.

If the target is not new, it is not possible to claim all oligonucleotides targeting the gene, but it is still possible to try to claim all oligonucleotides targeting a region of the mRNA. To this end, it is important to demonstrate that targeting the specific region of the mRNA confers a better effect than targeting other regions of the mRNA. Here is a theoretical example of a functional definition of a claim (e.g., see WO2010048228 [63] in Table 5 for a concrete example):

“An antisense oligonucleotide that reduces the expression and/or activity of [the target] and targets the nucleic acids sequence X to XX of the nucleic acid sequence as set for SEQ ID NO: X.”

Moreover, if the target is not new, it is still possible to claim specific oligonucleotides (with specific nucleic acid sequences) and thus write a structural definition of the oligonucleotides. The writing of this protection is quite common and easy to implement. The oligonucleotides for which protection is required are defined by their nucleic acid sequences, and these sequences are described in the patent application and in a sequence listing. ID numbers are attributed to each sequence (ex.: SEQ ID NO: X). Here is a theoretical example of a structural definition of a claim (e.g., see WO2014179627 [64] of Table 5 for a concrete example):

“An antisense oligonucleotide of gene X with a nucleic acid sequence as set forth in SEQ ID NO: 1.”

“Sequence listing example: SEQ ID NO: 1: acgcggtttattcggttaaa”

Of course, this kind of patent can also claim therapeutic methods using these specific oligonucleotides and pharmaceutical compositions containing them.

Another alternative to protect oligonucleotides is to claim a new scaffold. Indeed, inventors in the oligonucleotide field can be very creative and can develop new scaffolds of oligonucleotides. Generally, the claims are directed to a nucleic acid sequence that comprises specific nucleotides—for example, in a special order, with modifications or not and with a specific linker between each nucleic acid. In this case, the writer tries to achieve a broad scope of protection but also provides fallback positions if the broader claims are not accepted. Here is a theoretical example of a scaffold claim (e.g., see WO2013075035 [65] of Table 5 for a concrete example):

“An oligonucleotide with the following structure:

atcgatc-(x)N-aattccg

wherein (x) is any nucleic acid and N is comprised between 1 and 10,

wherein the nucleic acids of the oligonucleotide are linked by covalent bonds, and

wherein the oligonucleotide has no more than 60% guanosine nucleobases.”

Finally, another specificity concerning the protection of oligonucleotides is that modifications/improvements can also contribute to protecting a new object. Indeed, the inventors always seek to improve the efficiency of oligonucleotides—for example, in terms of their stability, their ability to enter in the cells, their half-life, etc. Thus, the modifications of the oligonucleotides, such as the addition of specific chemical group like GalNAc (see above) developed by Alnylam, can be protected itself but can also be used to improve the protection of the oligonucleotide already filed in a patent application (e.g., see WO2014179627 [64] of Table 5 for a concrete example). For example, a new patent application could be filed for the improved oligonucleotide (the oligonucleotide already patented but with the modification).

### 3.3. Prosecution in Front of the EPO and USPTO

As we have seen, different possibilities exist to protect oligonucleotide therapeutics. However, depending on the patent office, the invention is analyzed quite differently.

In Europe, at the EPO (www.epo.org, accessed on 20 November 2021), oligonucleotides are eligible, and it is usually not a problem to get a patent with a structural definition, if the patentability criteria are recognized (see Table 4). However, to obtain a broader scope on any oligonucleotides targeting a gene (functional definition), the gene and its targeting in a specific therapy have to be new (not disclosed in the prior art), as we saw above (see Table 4). Moreover, in terms of disclosure of the invention, the EPO is very attentive to the examples described in the patent application. Indeed, several examples with different oligonucleotides showing the effect are necessary.

In the same way, if a region of the mRNA is targeted or for a claim relating to a scaffold, several examples showing a better effect than targeting other regions of the mRNA or several examples with the specific scaffold are also necessary. In this case, and generally, the EPO accepts granting a broad claim. However, the degree of exemplification is higher in the case of the scaffold.

Finally, to obtain a patent that covers oligonucleotides with the same the specific modification (such as a chemical modification), it is necessary to provide experimental data describing different oligonucleotides with the modification.

Of course, in all these cases, the novelty and the inventive step are particularly studied by the EPO in regards to the prior art (see Table 4).

Another particularity with the EPO is that methods of treatment are not accepted because they are not patentable under article 53(c) of the European Patent Convention (EPC). However, the use of specific oligonucleotides for the treatment of a disease are accepted (first or second medical-use claims). Thus, a broad scope including, for example, all oligonucleotides targeting a gene or mRNA region for use in the treatment of a disease are also accepted if several examples are described in the patent application (see above) and, of course, if the novelty and the inventive step criteria are met.

In the United States, at the USPTO (www.uspto.gov, accessed on 20 November 2021), the situation is quite different. Indeed, obtaining functional definition or structural definition claims can be much more difficult.

Since the Myriad Genetics case and the decision of the Supreme Court on 13 June 2013, which ruled on the non-patentability of natural products [66], it is not possible to patent DNA without modification since it is considered a natural product. In this case, an oligonucleotide (which is DNA or RNA) without any modification is thus recognized as a product of nature and in theory is not eligible for patentability. However, an oligonucleotide with modifications can still be patentable [67]. An oligonucleotide with a modification, even a minor one, can be eligible with the USPTO. A structural definition of claim relating to an oligonucleotide with modifications is thus still possible. However, each case is carefully analyzed and in practice it is still recommended to provide the oligonucleotide without modification in the patent application since the use of it in a method of therapy is still eligible for patentability and does not fall under the scope of the Myriad decision [68]).

For a functional definition, the situation can also be difficult after a recent decision by the Federal Court of Appeal between Amgen and Sanofi [61]. Indeed, since this decision, it is not possible to obtain protection for all antibodies against a specific target. We can thus imagine that the USPTO will now probably apply this decision to oligonucleotides (and other products) against a target and that it will no longer be possible to obtain a broad claim (functional definition) on oligonucleotides directed at a specific target.

Finally, as seen above, oligonucleotides with modifications as such should be patentable in the USPTO if several examples show the effect of the modified oligonucleotides.

Of course, like for the EPO, the novelty and inventive step of the oligonucleotides are particularly studied in addition to their eligibility (see Table 4).

## 4. Discussion

The development of therapeutic oligonucleotides has required major evolutions to move from an attractive concept to the emergence of a new class of therapeutic agents. As with other drug-discovery platforms, this development has taken several decades and has experienced many ups and downs. Recent events such as the commercial success of nusinersen and the market access of GalNAc conjugates have marked important milestones in the recognition of oligonucleotides as therapeutic agents capable of competing with traditional modalities like small molecules and monoclonal antibodies. The initial promise of oligonucleotides was to be able to modulate any therapeutic target by modulating its expression level, which would make almost all therapeutic targets potentially druggable. Despite the many developments and successes described throughout this review, there is still a long way to go to make this promise a reality.

As of this writing, oligonucleotide technologies are still maturing and there are many opportunities to further improve them and create better drugs. The success of GalNAc conjugates has demonstrated the benefit of specifically targeting a given tissue [23]. Incorporating GalNAc conjugates into siRNAs and ASOs to direct a larger fraction of a dose to the liver greatly enhances the potency of these compounds and reduces the exposure of other tissues to the drug [69,70]. This allows for a reduction in the amount of drug to be injected and less frequent dosing, possibly reducing the side effects observed with higher doses of oligonucleotides. The great potency of GalNAc conjugates has largely motivated companies to develop oligonucleotides targeting hepatic genes and it is not surprising that almost half of the oligonucleotides identified in our study target the liver. However, systemic administration beyond the liver will require further research, innovation, and development. Several companies and academic laboratories have been concentrating efforts on identifying new ligands that enhance delivery of oligonucleotides to other tissues and cells. This requires the identification of tissue-specific membrane receptors and the development of ligands that can interact with a high affinity with these receptors and induce their internalization. Different classes of molecules can be used as ligands, including antibodies, aptamers, lipids, sugars, and peptides [11]. As an example, Ionis pharmaceuticals and AstraZeneca collaborate on several programs aimed at developing ligand-conjugated ASOs (LICAs) to enhance specific delivery in several tissues, including the heart, skeletal muscles, and pancreas. They reported that glucagon-like peptide-1 (GLP–1) peptide-conjugated ASOs concentrate in the beta cells of the pancreas [71].

As mentioned above, the CNS is one of the tissues most targeted by oligonucleotide therapeutics. However, due to the very restrictive BBB, which prevents most biomolecules and drugs from entering the CNS, these therapies require invasive intrathecal injections. As a result, only very severe pathologies have been addressed with oligonucleotides so far. The development of technologies allowing oligonucleotides to cross the BBB could broaden the spectrum of neurological pathologies addressable by oligonucleotides. Innovative technologies, including glucose-coated polymeric nanocarriers and cholesterol-functionalized DNA/RNA heteroduplexes, are currently under development to enable oligonucleotides to target the CNS following systemic administration [72,73].

For systemically administered of oligonucleotides, to date, subcutaneous injection is the preferred route of administration since nearly half of oligonucleotides (≈70% of those injected systemically) use this route of administration. Compared to the intravenous route, subcutaneous injection offers the advantage of potentially being self-administered, eliminating the need for patients to repeatedly visit a medical facility. Further improvements in terms of patient convenience could result from the development of formulations compatible with oral administration. Important developments are underway to allow oligonucleotide administration by this route, which is the one most used for drugs and the easiest for patients to use. A recent study led by AstraZeneca showed that an ASO conjugated to GalNAc and co-formulated in a tablet with sodium caprate can be delivered orally and absorbed by the liver, where it reduces the expression of its target gene (PCSK9) [50]. Sodium caprate is an activator of intestinal permeation that acts by opening tight epithelial junctions and/or disrupting the membrane to facilitate the transport of macromolecules into the bloodstream [74]. Once in the blood, the oligonucleotides can reach organs and specifically target the liver through GalNAcs. Since only a proportion of the molecules is able to pass into the blood after oral administration, it is important to use potent oligonucleotides. In the AstraZeneca study, the authors used a highly potent oligonucleotide containing cET modifications [50]. The chemical optimization of the oligonucleotides themselves to increase their potency, together with their co-formulation with permeation activators like sodium caprate or others, should allow the use of the oral route in clinics in the future beyond the use of this route only in a topical way to target the gastrointestinal tract.

To date, most oligonucleotides developed as drugs are gene-expression inhibitors (85% in our analysis). Expression-inhibiting oligonucleotides have proven to be efficient therapeutic agents when a target requires inhibition, and eight compounds including ASOs and siRNAs have reached the market [6,12,13,14,41,45,46,47]. It seems more challenging to activate the expression of a gene. However, when a therapeutic target requires activation, this can be achieved using oligonucleotides to target splicing events. Splicing modulators have proven their effectiveness, and five molecules acting by this mechanism have already entered the market [8,33,34,35,36]. The most common way to use exon skipping to restore the function of a mutated gene is to eliminate an exon containing a frameshift or a nonsense mutation to induce the production of a partially internally deleted but functional protein. This strategy is mainly used for DMD. An alternative approach for specific gene activation is the targeted augmentation of nuclear gene output (TANGO) method inspired by the mode of action of nusinersen and currently under development by Stoke Therapeutics. This strategy consists of targeting naturally occurring non-productive alternative splicing events, which results in premature termination codon generation and transcript degradation via nonsense-mediated decay (NMD) [75]. Splice-correcting ASOs target the sites of these non-productive alternative splicing products to promote the generation of the productive transcript isoform, thereby upregulating target gene expression and protein synthesis. An ASO developed based on this strategy, STK-001, is currently being evaluated in a phase II clinical trial for the treatment of Dravet syndrome [30]. An alternative to the use of splice modulators to increase gene expression is the use of saRNAs to directly activate transcription. The development of saRNAs as drugs is in a much earlier stage; for the time being only one molecule is being evaluated in a clinical trial [55] and none has reached the market. Despite the great interest that molecules capable of activating the expression of any gene may represent in terms of drug development, more results in patients will be necessary to conclude that a new class of drugs has emerged.

Lastly, we highlighted in this review that patenting oligonucleotides is of great importance for the companies exploiting this technology and that the examination of the patentability of the invention can differ from one country to another. Our recommendation as a tech transfer office is to provide for all aspects the broadest protection possible (notably, providing functional and structural claims, as explained above), because depending on the territory (e.g., Europe or United States), the protection may not be the same in the end.

In conclusion, oligonucleotides have demonstrated their ability to produce major therapeutic benefits for patients and further developments will likely lead to even better drugs coming to the market in the near future.

## Figures and Tables

**Table 1:** Antisense oligonucleotides (ASOs) approved or in clinical development.

Drug Name	Target Gene	Mode of Action	Therapy Area	Latest Stage of Development	Company
Nusinersen	SMN2	Splicing modulation	Neurology	Marketed	Biogen
Eteplirsen	DMD	Splicing modulation	Muscular disorders	Marketed	Sarepta Therapeutics
Inotersen	TTR	Expression inhibition	Metabolic disorders	Marketed	Akcea Therapeutics
Viltolarsen	DMD	Splicing modulation	Muscular disorders	Marketed	NS Pharma
Casimersen	DMD	Splicing modulation	Muscular disorders	Marketed	Sarepta Therapeutics
Golodirsen	DMD	Splicing modulation	Muscular disorders	Marketed	Sarepta Therapeutics
Mipomersen	APOB	Expression inhibition	Metabolic disorders	Marketed	Kastle Therapeutics
Volanesorsen	APOC3	Expression inhibition	Metabolic disorders	Marketed	Akcea Therapeutics
Fomivirsen	CMV virus IE2	Expression inhibition	Infectious disease	Marketed and withdrawn	Novartis
Aganirsen	IRS1	Expression inhibition	Ophthalmology and metabolic disorders	Phase III	Gene Signal
Alicaforsen	ICAM1	Expression inhibition	Gastrointestinal	Phase III	Atlantic Healthcare
Eplontersen	TTR	Expression inhibition	Metabolic disorders	Phase III	Akcea Therapeutics
ION-363	FUS	Expression inhibition	Neurology	Phase III	Ionis Pharmaceuticals
Olezarsen	APOC3	Expression inhibition	Cardiovascular and metabolic disorders	Phase III	Akcea Therapeutics
Pelacarsen	LPA	Expression inhibition	Cardiovascular and metabolic disorders	Phase III	Novartis
Sepofarsen	CEP290	Splicing modulation	Ophthalmology	Phase III	ProQR Therapeutics
Tofersen	SOD1	Expression inhibition	Neurology	Phase III	Biogen
Tominersen	HTT	Expression inhibition	Neurology	Phase III	Roche
Trabedersen	TGFB2	Expression inhibition	Oncology	Phase III	Oncotelic
Zilganersen	GFAP	Expression inhibition	Neurology	Phase III	Ionis Pharmaceuticals
ASM-8	CCR3 and CSF2RB	Expression inhibition	Respiratory	Phase II	Pharmaxis
Atesidorsen	GHR	Expression inhibition	Hormonal disorders	Phase II	Antisense Therapeutics
ATL-1102	ITGA4	Expression inhibition	Neurology and muscular disorders	Phase II	Antisense Therapeutics
AZD-8233	PCSK9	Expression inhibition	Metabolic disorders	Phase II	AstraZeneca
AZD-8701	FOXP3	Expression inhibition	Oncology	Phase II	AstraZeneca
Bepirovirsen	Viral HBV	Expression inhibition	Infectious disease	Phase II	Ionis Pharmaceuticals
BIIB-080	MAPT	Expression inhibition	Neurology	Phase II	Biogen
Cepadacursen	PCSK9	Expression inhibition	Metabolic disorders	Phase II	Civi Biopharma
Cimderlirsen	GHR	Expression inhibition	Hormonal disorders	Phase II	Ionis Pharmaceuticals
CODA-001	GJA1	Expression inhibition	Ophthalmology	Phase II	Eyevance Pharmaceuticals
Danvatirsen	STAT3	Expression inhibition	Oncology	Phase II	AstraZeneca
Donidalorsen	KLKB1	Expression inhibition	Immunology and infectious disease	Phase II	Ionis Pharmaceuticals
DYN-101	DYN2	Expression inhibition	Muscular disorders	Phase II	Dynacure
GTX-102	UBE2A	Expression inhibition	Neurology	Phase II	GeneTx Biotherapeutics
ION-224	DGAT2	Expression inhibition	Gastrointestinal	Phase II	Ionis Pharmaceuticals
ION-253	Undisclosed	Expression inhibition	Gastrointestinal	Phase II	Johnson & Johnson
ION-464	SNCA	Expression inhibition	Neurology	Phase II	Ionis Pharmaceuticals
ION-541	ATXN2	Expression inhibition	Neurology	Phase II	Ionis Pharmaceuticals
ION-859	LRRK2	Expression inhibition	Neurology	Phase II	Ionis Pharmaceuticals
IONIS-AGTLRx	AGT	Expression inhibition	Cardiovascular	Phase II	Ionis Pharmaceuticals
IONIS-FB-LRx	CFB	Expression inhibition	Genitourinary system and ophthalmology	Phase II	Ionis Pharmaceuticals
IONIS-FXILRx	F11	Expression inhibition	Cardiovascular, hematology, and genitourinary system	Phase II	Ionis Pharmaceuticals
IONIS-GCGRRx	GCGR	Expression inhibition	Metabolic disorders	Phase II	Ionis Pharmaceuticals
IONIS-HBVLRx	Viral HBV	Expression inhibition	Infectious disease	Phase II	Ionis Pharmaceuticals
IONIS-PKKRx	KLKB1	Expression inhibition	Neurology	Phase II	Ionis Pharmaceuticals
IONISAR-2.5Rx	AR	Expression inhibition	Oncology	Phase II	Ionis Pharmaceuticals
IONISENAC-2.5Rx	SCNN1A	Expression inhibition	Respiratory	Phase II	Ionis Pharmaceuticals
IONISTMPRSS-6LRx	TMPRSS6	Expression inhibition	Hematology	Phase II	Ionis Pharmaceuticals
ISTH-0036	TGFB2	Expression inhibition	Ophthalmology	Phase II	Isarna Therapeutics
NS-089	DMD	Splicing modulation	Muscular disorders	Phase II	Nippon Shinyaku
Prexigebersen	GRB2	Expression inhibition	Oncology	Phase II	Bio-Path Holdings
QR-1123	RHO	Expression inhibition	Ophthalmology	Phase II	ProQR Therapeutics
QRX-421a	USH2A	Splicing modulation	Ophthalmology	Phase II	ProQR Therapeutics
Renadirsen	DMD	Splicing modulation	Muscular disorders	Phase II	Daiichi Sankyo
SRP-5051	DMD	Splicing modulation	Muscular disorders	Phase II	Sarepta Therapeutics
STK-001	SCN1A	Splicing modulation	Neurology	Phase II	Stoke Therapeutics
Vupanorsen	ANGPTL3	Expression inhibition	Metabolic disorders	Phase II	Pfizer
WVE-003	HTT	Expression inhibition	Neurology	Phase II	Wave Life Sciences
WVE-004	C9orf72	Expression inhibition	Neurology	Phase II	Wave Life Sciences
WVEN-531	DMD	Splicing modulation	Muscular disorders	Phase II	Wave Life Sciences

**Table 2:** Small interfering RNAs (siRNAs) approved or in clinical development.

Drug Name	Target Gene	Mode of Action	Therapy Area	Latest Stage of Development	Company
Patisiran	TTR	Expression inhibition	Metabolic disorders	Marketed	Alnylam Pharmaceuticals
Givosiran	ALAS1	Expression inhibition	Metabolic disorders	Marketed	Alnylam Pharmaceuticals
Inclisiran	PCSK9	Expression inhibition	Cardiovascular and metabolic disorders	Marketed	Novartis
Lumasiran	HAO1	Expression inhibition	Genitourinary system	Marketed	Alnylam Pharmaceuticals
Vutrisiran	TTR	Expression inhibition	Cardiovascular and metabolic disorders	Pre-registration	Alnylam Pharmaceuticals
Fitusiran	SERPINC1	Expression inhibition	Hematology	Phase III	Sanofi
Nedosiran	LDHA	Expression inhibition	Genitourinary system	Phase III	Dicerna Pharmaceuticals
QPI-1007	CASP2	Expression inhibition	Ophthalmology	Phase III	Quark Pharmaceuticals
Teprasiran	TP53	Expression inhibition	Immunology	Phase III	Quark Pharmaceuticals
Tivanisiran	TRPV1	Expression inhibition	Ophthalmology	Phase III	Sylentis
AB-729	HBsAg	Expression inhibition	Infectious disease	Phase II	Arbutus Biopharma
ALNAAT-02	SERPINA1	Expression inhibition	Gastrointestinal and metabolic disorders	Phase II	Alnylam Pharmaceuticals
ARO-HSD	HSD17B13	Expression inhibition	Gastrointestinal	Phase II	Arrowhead Pharmaceuticals
AROANG-3	ANGPTL3	Expression inhibition	Metabolic disorders	Phase II	Arrowhead Pharmaceuticals
AROAPOC-3	APOC3	Expression inhibition	Metabolic disorders	Phase II	Arrowhead Pharmaceuticals
Bamosiran	ADRB2	Expression inhibition	Ophthalmology	Phase II	Sylentis
Belcesiran	SERPINA1	Expression inhibition	Gastrointestinal	Phase II	Dicerna Pharmaceuticals
BMS-986263	SERPINH1	Expression inhibition	Gastrointestinal and respiratory	Phase II	Bristol-Myers Squibb
Cemdisiran	C5	Expression inhibition	Genitourinary system	Phase II	Alnylam Pharmaceuticals
Fazirsiran	SERPINA1	Expression inhibition	Metabolic disorders	Phase II	Arrowhead Pharmaceuticals
JNJ-3989	viral HBV	Expression inhibition	Infectious disease	Phase II	Arrowhead Pharmaceuticals
MT-5745	CHST15	Expression inhibition	Gastrointestinal	Phase II	Mitsubishi Tanabe Pharma
Olpasiran	LPA	Expression inhibition	Cardiovascular	Phase II	Amgen
OLX-101	CTGF	Expression inhibition	Dermatology	Phase II	Hugel/OliX Pharmaceuticals
RG-6346	HBsAg	Expression inhibition	Infectious disease	Phase II	Dicerna Pharmaceuticals
siG-12D-LODER	KRAS	Expression inhibition	Oncology	Phase II	Silenseed
SR-063	AR	Expression inhibition	Oncology	Phase II	Suzhou Ribo Life Sciences
STP-705	PTGS2/TGFB1	Expression inhibition	Oncology and dermatology	Phase II	Sirnaomics
VIR-2218	HBsAg	Expression inhibition	Infectious disease	Phase II	Alnylam Pharmaceuticals
Zilebesiran	AGT	Expression inhibition	Cardiovascular	Phase II	Alnylam Pharmaceuticals

**Table 3:** Oligonucleotides other than ASOs and siRNAs in clinical development.

Drug Name	Target Gene	Mode of Action (Type of Compound)	Therapy Area	Latest Stage of Development	Company
Lademirsen	MIR21	Expression inhibition (miRNA inhibitor)	Genitourinary system	Phase II	Sanofi
MTL-CEBPA	CEBPA	Expression activation (saRNA)	Oncology	Phase II	Mina Therapeutics
remlarsen	MIR29B1	Expression activation (miRNA mimic)	Dermatology	Phase II	Miragen Therapeutics

**Table 4:** How to justify the novelty and the inventive step of an oligonucleotide with regard to prior art.

Is the Target Known?	Is There at Least One Oligonucleotide against This Target?	Novelty?		Inventive Step?	
No	No	Europe	Yes, because the target is new (functional definition possible; however, in the US the oligonucleotide must have a modification to be eligible).	Europe	Yes, because the target is new.
		United States		United States	Yes, but the USPTO could refuse a patent on the target (comparison to the decision between Amgen v. Sanofi [61] relating to the protection of antibodies).
Yes	No	Europe	Yes, because it is the first oligonucleotide used in therapy (however, in the US, the oligonucleotide must have a modification to be eligible and a functional definition is not possible).	Europe	Yes, if the oligonucleotide has a particular functional characteristic.
		United States		United States	
Yes	Yes	Europe	Yes, if the oligonucleotide has a different sequence/structure (e.g., with modifications) than oligonucleotides of the prior art (However, in the US, the oligonucleotide must have a modification to be eligible and a functional definition is not possible).	Europe	No, unless the oligonucleotide has an unexpected or new property compared to the oligonucleotides disclosed in the prior art (e.g., addition of a new modification, surprising effect) or if the oligonucleotide targets a particular region (e.g., a particular region of a gene).
		United States		United States	

**Table 5:** Types of claims in practice.

Type of Claim	Drug Name	Latest Stage of Development	Patent Application	Claims
Functional definition	Casimersen	Marketed	WO2006000057 [62]	1. An antisense molecule capable of binding to a selected target site to induce exon skipping in the dystrophin gene, as set forth in SEQ ID NO: 1 to 202.
	Patisiran	Marketed	WO2010048228 [63]	1. A double-stranded ribonucleic acid (dsRNA) for inhibiting expression of transthyretin (TTR), wherein said dsRNA comprises a sense strand and an antisense strand, the antisense strand comprising a region complementary to a part of an mRNA encoding transthyretin (TTR), wherein said region of complementarity is less than 30 nucleotides in length and the antisense strand comprises 15 or more contiguous nucleotides of SEQ ID NO:170, SEQ ID NO:450, SEQ ID NO:730, or SEQ ID NO:1010.
Structural definition	Inotersen	Marketed	WO2014179627 [64]	1. A compound comprising a modified oligonucleotide and a conjugate group, wherein the modified oligonucleotide consists of eight to 80 linked nucleosides and has a nucleobase sequence at least 85%, 90%, 95%, or 100% complementary to SEQ ID NO: 2 encoding transthyretin (TTR).
Scaffold claim	Vutrisiran	Pre-registration	WO2013075035 [65]	1. A double-stranded RNAi agent comprising a sense strand complementary to an antisense strand, wherein said antisense strand comprises a region complementary to part of an mRNA encoding transthyretin (TTR), wherein each strand has about 14 to about 30 nucleotides, wherein said double-stranded RNAi agent is represented by formula (III): sense: 5′ np -Na -(X X X);-Nb -Y Y Y -Nb -(Z Z Z)j -Na-nq 3′ antisense: 3′ np’-Na’-(X’X’X’)k-Nb’-Y’Y’Y’-Nb’-(Z’Z’Z’)i-Na’- nq’ 5′ (III) wherein i, j, k, and 1 are each independently 0 or 1; p, p’, q, and q’ are each independently 0–6; each Na and Na’ independently represents an oligonucleotide sequence comprising 0–25 nucleotides that are either modified or unmodified or combinations thereof, each sequence comprising at least two differently modified nucleotides; each Nb and Nb’ independently represents an oligonucleotide sequence comprising 0–10 nucleotides that are either modified or unmodified or combinations thereof; each np, np’, nq, and nq’ independently represents an overhang nucleotide; XXX, YYY, ZZZ, Χ’Χ’Χ’, ΥΎΎ’, and Z’Z’Z’ each independently represent one motif of three identical modifications on three consecutive nucleotides; modifications on Nb differ from the modification on Y and modifications on Nb’ differ from the modification on Y’; and wherein the sense strand is conjugated to at least one ligand.

## Data Availability

Data were collected from GlobalData (https://www.globaldata.com/, accessed on 20 November 2021), Clinical Trials (https://clinicaltrials.gov/, accessed on 20 November 2021), and Pubmed (https://pubmed.ncbi.nlm.nih.gov/, accessed on 20 November 2021).
